# A novel method for evaluating antibody-dependent cell-mediated cytotoxicity by flowcytometry using cryopreserved human peripheral blood mononuclear cells

**DOI:** 10.1038/srep19772

**Published:** 2016-01-27

**Authors:** Makiko Yamashita, Shigehisa Kitano, Hiroaki Aikawa, Aya Kuchiba, Mitsuhiro Hayashi, Noboru Yamamoto, Kenji Tamura, Akinobu Hamada

**Affiliations:** 1 Department of Clinical Pharmacology, National Cancer Center Research Institute, 5-1-1 Tsukiji, Chuo-ku, Tokyo 104-0045, Japan; 2 Department of Experimental Therapeutics, National Cancer Center Hospital, 5-1-1 Tsukiji, Chuo-ku, Tokyo 104-0045, Japan; 3 Exploratory Oncology Research and Clinical Trial Center, National Cancer Center, 5-1-1 Tsukiji, Chuo-ku, Tokyo 104-0045, Japan; 4 Division of Biostatistics, Center for Research Administration and Support, National Cancer Center, 5-1-1 Tsukiji, Chuo-ku, Tokyo 104-0045, Japan; 5 Division of Breast and Medical Oncology, National Cancer Center, 5-1-1 Tsukiji, Chuo-ku, Tokyo 104-0045, Japan

## Abstract

Analyzing the cytotoxic functions of effector cells, such as NK cells against target cancer cells, is thought to be necessary for predicting the clinical efficacy of antibody-dependent cellular cytotoxicity (ADCC) -dependent antibody therapy. The ^51^Cr release assay has long been the most widely used method for quantification of ADCC activity. However, the reproducibilities of these release assays are not adequate, and they do not allow evaluation of the lysis susceptibilities of distinct cell types within the target cell population. In this study, we established a novel method for evaluating cytotoxicity, which involves the detection and quantification of dead target cells using flowcytometry. CFSE (carboxyfluorescein succinimidyl ester) was used as a dye to specifically stain and thereby label the target cell population, allowing living and dead cells, as well as both target and effector cells, to be quantitatively distinguished. Furthermore, with our new approach, ADCC activity was more reproducibly, sensitively, and specifically detectable, not only in freshly isolated but also in frozen human peripheral blood mononuclear cells (PBMCs), than with the calcein-AM release assay. This assay, validated herein, is expected to become a standard assay for evaluating ADCC activity which will ultimately contribute the clinical development of ADCC dependent-antibody therapies.

Recently, there has been rapid progress in the field of clinical immunotherapy. The recent confirmation of the clinical efficacies of several immunotherapeutic drugs in patients with cancers has promoted the development of this treatment strategy. In particular, the use of monoclonal antibodies (mAbs) for cancer therapy is one of the most successful and important strategies for treating cancer patients^1^. Such mAbs can kill tumor cells by (1) blocking the function of the target molecule, (2) mediating the delivery of cytotoxic drugs, (3) affecting the tumor vasculature or stroma, and/or (4) triggering immune-mediated cell killing mechanisms. The development of a valid assay for monitoring currently relevant immune responses remains one of the greatest hurdles to overcome in this field of research^2^.

“Trastuzumab”, a humanized mAb directed against the extracellular domain of the HER2 receptor, is among the most well known antibody-based drugs. For over 10 years, Trastuzumab has been widely used in the treatment of HER2-positive breast cancers. It triggers immune-mediated responses against HER2-overexpressing cells via antibody-dependent cellular cytotoxicity (ADCC). In approximately 20% of breast cancer patients with metastases and whose tumors overexpress the HER2/neu protein^3^, Trastuzumab-based chemotherapy resulted in a modest increase in survival^4^. Although response rates to Trastuzumab-based chemotherapy of HER2-overexpressing breast cancers can exceed 50%^5^, the vast majority of patients will eventually experience disease progression, despite ongoing Trastuzumab therapy^3^. Previous studies showed impaired stimulation of the ADCC response to be associated with Trastuzumab resistance. One patient who had a pathologic complete response reportedly experienced very intense ADCC, whereas four others who had partial responses showed intermediate ADCC^6^,^7^. Complete or partial remission in patients treated with neoadjuvant Trastuzumab correlated with tumor infiltration of immune cells and higher *in vitro* ADCC activity in a lysis assay^8^. These observations indicated lack of responsiveness to Trastuzumab to be associated with inability to mount an ADCC response. It is important to characterize the immune profiles of responders, and to understand those of non-responders, potentially yielding valuable information, which might reveal the diversity of mechanisms controlling antitumor immunity^9^.

ADCC is a result of Fc-gamma receptor (FcγR) mediated interaction with effector immune cells such as natural killer (NK) cells, macrophages and granulocytes. The binding of FcγR to the Fc domain induces the release of both granzyme and perforin from effector cells, leading to target cell lysis and Fc-dependent tumor cell phagocytosis^10^. It is necessary to analyze these effector functions against target cancer cells to clinically evaluate the efficacy of antibody-immunotherapy. The most widely used assay for quantification of ADCC is the conventional ^51^Cr (chromium) release assay^11^,^12^. The ^51^Cr release assay has long been the standard technique for measuring cell-mediated cytotoxicity. Though this method has the benefits of being reproducible and relatively easy to perform, it has several drawbacks: (1) only semi-quantitative data are obtained unless limiting dilution assays are performed; (2) sensitivity is relatively low; (3) there is poor labeling of some target cell lines; (4) high spontaneous release from some target cell lines occurs; and (5) there are biohazard and disposal problems associated with radioisotope usage^1^,^13^. Recently, alternative assays (including lactate dehydrogenase (LDH), the 3-(4,5-dimethylthiazol-2-yl)-2,5-diphenyl tetrazolium bromide (MTT), and calcein-acetoxymethyl (calcein-AM) release) have been employed, in efforts to avoid exposure to radioactive materials from ^51^Cr labeling, due to concerns about the handling and disposal of radioactive materials. Moreover, a number of flowcytometric methods for measuring cell-mediated immunity, particularly those based on uptake of 7-amino-actinomycin D (7-AAD) or propidium iodide (PI), and Annexin V binding have been suggested as alternatives to the ^51^Cr release assay. However, these release assays are known to have poor reproducibility, not allowing evaluation of the lysis susceptibilities of distinct cell types within the target cell population^12^,^14^.

Cytotoxic reactions have not been adequately investigated in individual cancer patients given antibody therapy with ADCC activity. It is important to develop a standard analysis allowing routine measurement of ADCC activity. We established a novel ADCC assay method for measuring cytotoxicity. This assay detects and quantifies dead target cells using flowcytometry. With our method, living and dead target and effector cells can be distinguished based on differential staining by fluorescent dyes. Moreover, this assay is capable of assessing the cytotoxicity induced by frozen peripheral blood mononuclear cells (PBMCs).

## Results

### Flowcytometric analysis of cell surface molecule expressions on target and effector cell lines

**Figure 1:**
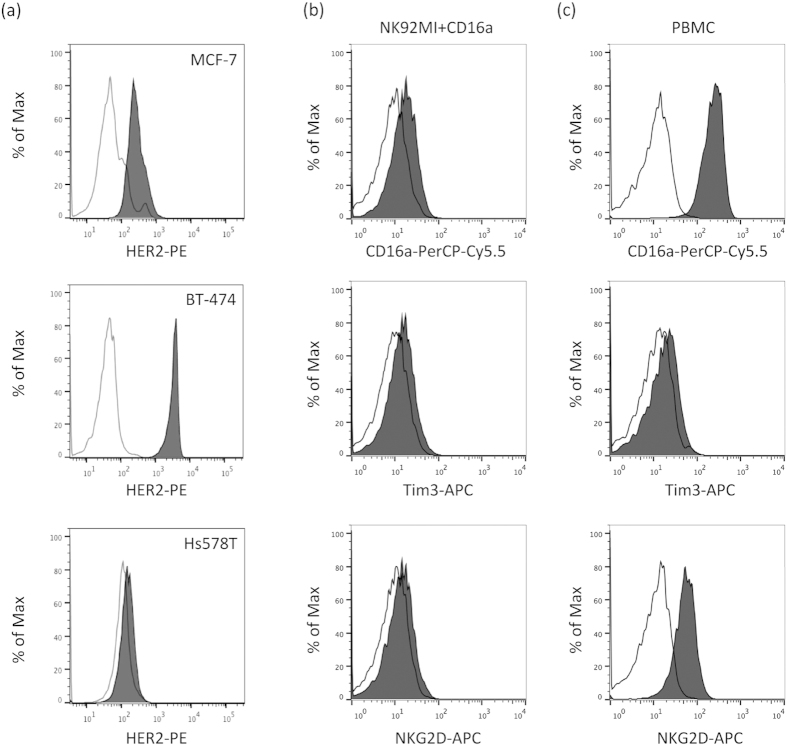
Phenotypic analyses of three human breast cancer cell lines and NK cells. (**a**) Expression levels of the cell surface HER2 (black filled histogram) on human breast cancer cell lines measured using flowcytometry. The histograms in black lines represent the isotype control. (**b,c**) NK92MI + CD16a cells (**b**) or NK cells in freshly isolated healthy human PBMCs (**c**) were stained with anti-CD16 antibody (upper panel, black-filled histogram), anti-Tim3 antibody (middle panel, black-filled histogram) and anti-NKG2D antibody (lower panel, black-filled histogram). Respective isotype controls are shown as black lines in the histograms in both panels.

We used three human breast cancer cell lines, BT-474, MCF-7 and Hs578T, as the target cells, and one human NK cell line as the effector cells. We first measured the levels of HER2/neu expression on the three breast cancer cell lines. FACS analysis showed that BT-474 cells expressed a high level of HER2, while that on MCF-7 cells was moderate, and the Hs578T cells showed no expression (Fig. 1a). We next selected the BT-474 cell line as a target for examining Trastuzumab-mediated ADCC. On the other hand, NK cells are well known to be a major mediator of ADCC. We thus also employed PBMCs or the human NK cell line NK-92MI, exogenously expressing a 158V variant of FcγRIIIa (CD16a; NK92MI + CD16a), as effector cells. As previously demonstrated, the NK92MI + CD16a cell line, but not the NK92MI + mock, was capable of mediating ADCC 15. Moreover, this NK92MI + CD16a cell line or NK in PBMCs was found to express CD16a, as well as NK activation marker molecules such as NKG2D and Tim3, on the cell surface (Fig. 1b,c). The ADCC was analyzed using BT-474 as target cells and PBMCs or NK92MI + CD16a as effector cells, and Trastuzumab-mediated ADCC activity was then measured.

### Trastuzumab-mediated cytotoxicity against breast cancer cells in the NK92-MI cell line

**Figure 2:**
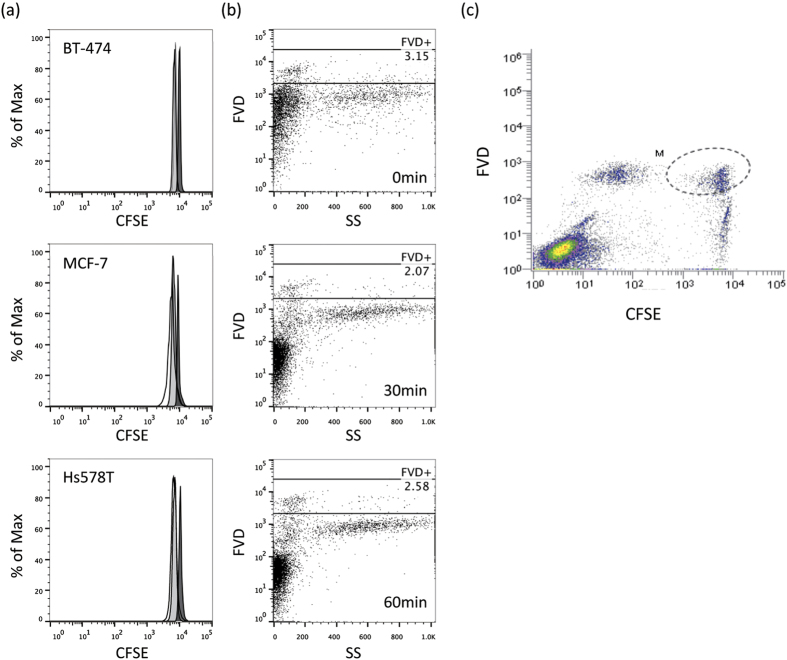
Staining strategy of the new flowcytometric assay. (**a**) Breast cancer cell lines, BT-474, MCF-7 and Hs578T, were stained with CFSE, and the intensity was then measured by flowcytometry, soon after staining (black filled histogram), after an overnight incubation (gray-filled histogram) and after 2 days of incubation (open histogram). (**b**) Human PBMCs were stained with FVD (1:1000 dilution, at RT for 20 min.), PI (2 μg/mL, at RT for 10 min.), or 7-AAD (5 μg/mL, at RT for 10 min.), and the dead cells (%) were then measured by flowcytometry. (**c**) Data obtained with the new flowcytometric analysis method. CFSE^ + ^cells are target cells, and FVD^ + ^cells are dead cells, such that dead target cells are represented by the CFSE^ + ^FVD^ + ^area.

We used CFSE as a dye to specifically stain the target cell population. CFSE spontaneously and irreversibly binds intracellular proteins. In all of the assays, the CFSE level was maintained essentially at an intensity of 10^4^, as compared to that of CFSE channel, in each cell population, although very slightly decreases were observed after incubation (Fig. 2a). On the other hand, for dead cell staining we employed FVD, as described above. Unlike 7-AAD and PI, staining with FVD is based on stable labeling (Fig. 2b and Figure S1), allowing quantitative analysis of the number of CFSE-labeled target cells having undergone apoptosis. As shown in Fig. 2c, target cells are represented by the CFSE^+^ area, and death of target cells as the CFSE^ + ^FVD^+^ area (encircled by the dashed line). We evaluated ADCC activity based on the number of dead target cells, as represented by the CFSE^+^ FVD^+^ area.

**Figure 3:**
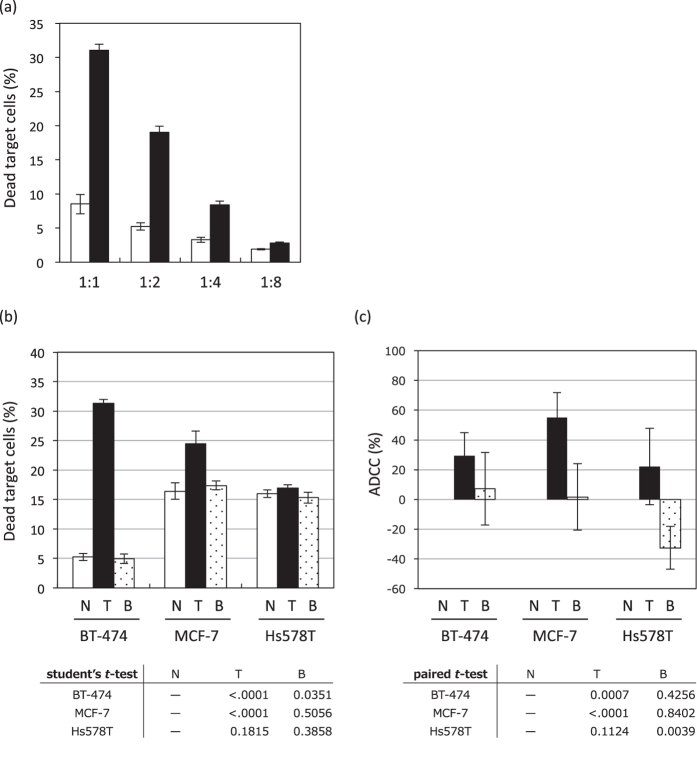
Assessment of the ADCC bioassay using the NK cell line. (**a**) The BT-474 as target cells were incubated overnight with NK92MI + CD16a as effector cells at various E:T ratios without (white bar) or with 10 μg/mL of Trastuzumab (black bar). (**b,c**) The three human breast cancer cell lines, expressing different HER2 levels, serving as target cells were incubated overnight with the NK92 + CD16a as effector cells, at an E:T ratio of 1:1, without (white bar) or with 10 μg/mL of Trastuzumab (black bar) or Bevacizumab (dot bar) using the flowcytometric assay (**b**) or calcein-release assay (**c**). The results of the analyses of dead cells (%) and ADCC (%) are presented as mean values ± SD of at least three experiments.

Initially, the optimal E:T cell ratios were determined in the presence of 10 μg/mL of Trastuzumab, rather than Bevacizumab. As shown in Fig. 3a, dead target cells were increased only in the presence of Trastuzumab, at all E:T ratios examined, though cytotoxicity was more effectively induced at the low ratio of 1:1, an E:T ratio at which Trastuzumab was three times more cytotoxic than Bevacizumab.

Moreover, to investigate specificity for HER2 (the target molecule of Trastuzumab), we used the three aforementioned human breast cancer cell lines, which have different HER2 expression levels (Fig. 1a). These cell lines stained with CFSE, and were co-incubated with NK92MI + CD16a at an E:T ratio of 1:1 in the presence of 10 μg/mL of mAb. In Hs578T, the cell line showing no HER2 expression, the number of dead target cells was not increased even with Trastuzumab treatment (Fig. 3b). Moreover, in MCF-7 and BT-474, the HER2 expressing cell lines, the number of dead target cells was increased by treatment with Trastuzumab but not by that with Bevacizumab (Fig. 3b). The cytotoxic effect of Trastuzumab was more effectively induced on the BT-474 than on the MCF-7 cell line, observations consistent with the HER2 expression levels on the surfaces of these cells. On the other hand, while the calcein-release assay, one of the most widely used methods for detecting ADCC activity, was found to be capable of detecting Trastuzumab-induced ADCC (*P*-values < 0.001) in the HER2-expressing cell lines BT-474 and MCF-7, the ADCC activity did not correlate with the HER2 expression levels (Fig. 3c). In flowcytometric assay, Trastuzumab alone exerted no significant effect on the survival of target cells (Figure S2), suggesting that this assay can detect ADCC induced by Trastuzumab.

### Flowcytometric assay is more sensitive than calcein-AM- release assay

**Figure 4:**
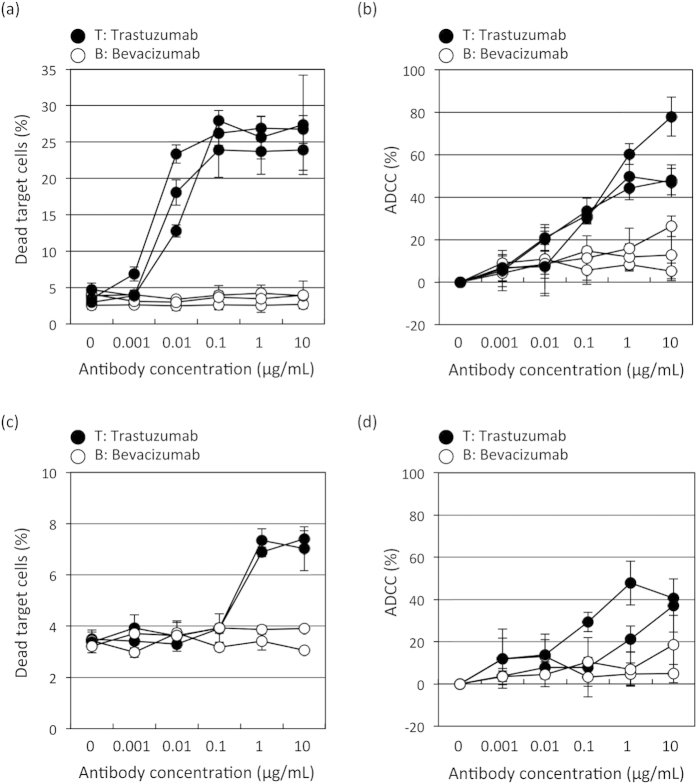
Comparison of the new flowcytometric and calcein-release assay. The flowcytometric (**a**,**c**) and calcein-release (**b,d**) assays were performed on different days, independently, to assess their reliability and reproducibility for evaluating ADCC, using NK92MI + CD16a (**a,b**) or PBMCs freshly isolated from the same healthy donor on different days (**c,d**). The results of the analyses of dead target cells (%) and ADCC (%) are presented as mean values ± SD for each of the experiments.

To evaluate the applicability of our novel flowcytometric assay, we compared ADCC detection abilities between our new method and the calcein-release assay. The dead target cells (%) in the flowcytometric assay and the ADCC (%) in the calcein-release assay using NK92MI + CD16a as effector cells are summarized in Fig. 4a,b (and Figure S3; fluorescence value), and Table S1 shows the means and standard deviations (SDs) of three independent experiments. In both assays, the ADCC activity measurement values tended to rise with increasing Trastuzumab concentrations. We compared the five concentrations with the non-treated (0 μg/mL) condition for each antibody. In Trastuzumab-treated cells, the means of the dead target cells (%) in the flowcytometric assay at 0.01 μg/mL or more are significantly different from that in the non-treated cells (all *P*-value <0.01, Fig. 4a and Table S2). There are no differences in the means of the dead target cells (%) between the non-treated cell populations and those at any of the five concentrations of Bevacizumab tested (Fig. 4a and Table S2). On the other hand, the means of the ADCC (%) at any of the five Trastuzumab concentrations are significantly different from the non-treated mean ADCC (all *P*-values <0.01, Fig. 4b and Table S2). However, significant differences are also observed in the means of ADCC (%) between the non-treated condition and Bevacizumab concentrations of 0.1 μg/mL or more (Fig. 4b and Table S2). Moreover, when using PBMCs as the effector cells (summarized in Table S3), the means of the dead target cells (%) in the flowcytometric assay at Bevacizumab concentrations of 1 to10 μg/mL are significantly different from that in the non-treated condition (*P*-values <0.01, Fig. 4c and Table S4). There are no differences in the means of the dead target cells (%) between the non-treated condition and any of the five concentrations of Bevacizumab tested (Fig. 4c and Table S4). On the other hand, except for the treatment with 10 μg/mL Trastuzumab, there are no differences in the means of the ADCC (%) (Fig. 4d and Table S4). In 10 μg/mL Trastuzumab-treated cells, the means of the ADCC (%) in the calcein-release assay are significantly different from that in the non-treated condition (*P*-value = 0.0004, Fig. 4d and Table S4). However, there are no differences in the means of ADCC (%) in the 10 μg/mL Trastuzumab-treated and 10 μg/mL Bevacizumab-treated cells, indicating that the calcein-release assay cannot detect Trastuzumab specific ADCC activity (Fig. 4d and Table S4). These results suggest that our new method detects ADCC activity more sensitively and specifically than the conventionally-used calcein-release assay.

### Evaluation of healthy-donor PBMC-specific ADCC

Our new ADCC assay was applied to human PBMCs freshly isolated from the blood of healthy volunteer donors.

**Figure 5:**
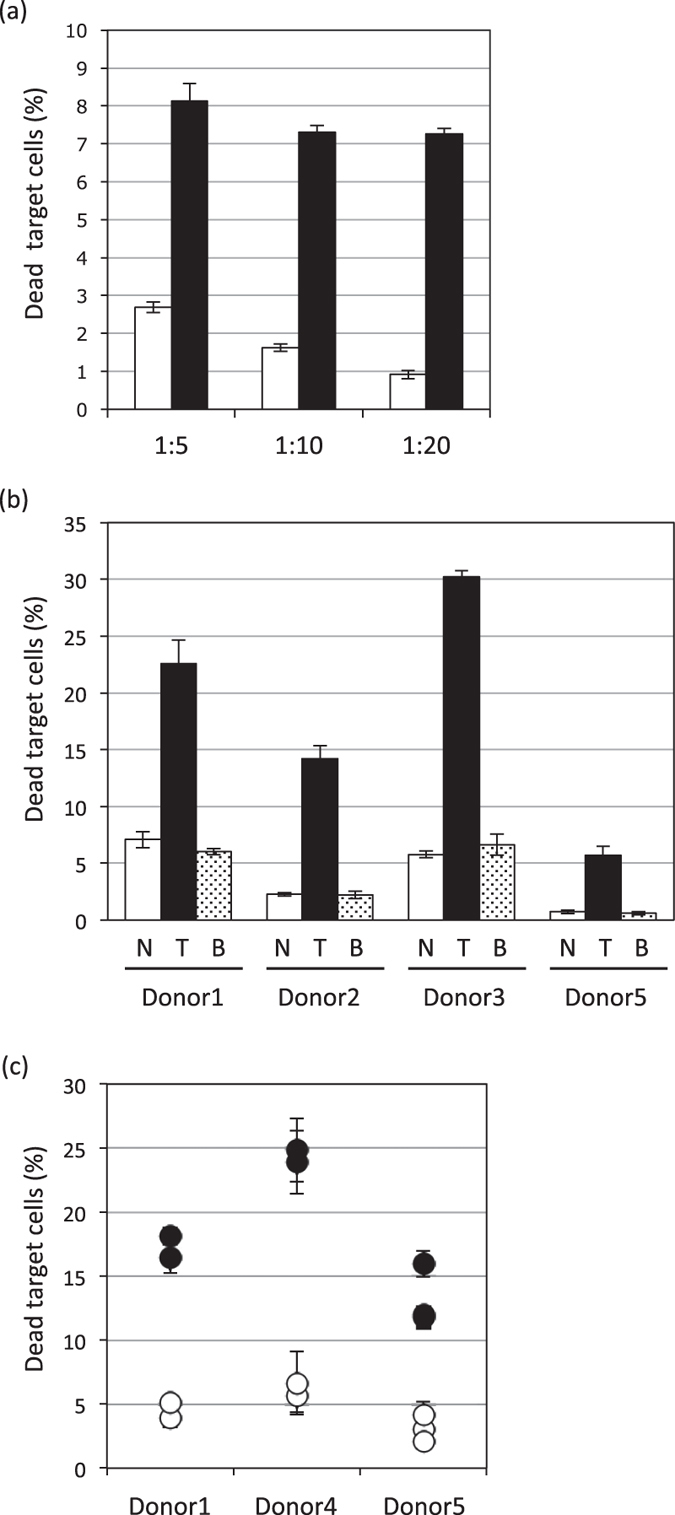
Evaluation of healthy-donor specific ADCC. (**a**) BT-474 serving as target cells were incubated overnight with PBMCs, serving as effector cells, freshly isolated from healthy volunteers. Various E:T ratios, without (white bar) or with 10 μg/mL of Trastuzumab (black bar), were employed. (**b**) PBMCs, freshly isolated from four healthy volunteers, were used as effector cells and were found to be capable of inducing individual ADCC activities against BT-474 as target cells, when incubated overnight without (white bar) or with 10 μg/mL of Trastuzumab (black bar) or Bevacizumab (dot bar). (**c**) The individual ADCC activities, determined every 1~2 months using freshly isolated PBMCs from healthy volunteers, were measured employing our flowcytometric assay with (black circles) or without (white circles) Trastuzumab. The results of the analyses of dead target cells (%) are presented as mean values ± SD for each of the experiments.

First, to determine optimal E:T cell ratios for human PBMCs, assays were performed in the presence of 10 μg/mLof Trastuzumab (Fig. 5a). The cytotoxicity induced by Trastuzumab was sufficient even at low E:T ratios after 24h of treatment, i.e. we were able to carry out this assay even with small volumes of blood. To determine the sensitivity of the new assay, PBMCs from four healthy donors were cultivated at an E:T ratio of 4:1 for 24h. As shown in Fig. 5b, our method allows the identification of individual differences among donors. Moreover, very stable individual ADCC activities were demonstrated using different blood samples isolated on other days from the same donors (Fig. 5c).

### Assessment of the new ADCC assay using frozen human PBMCs

**Figure 6:**
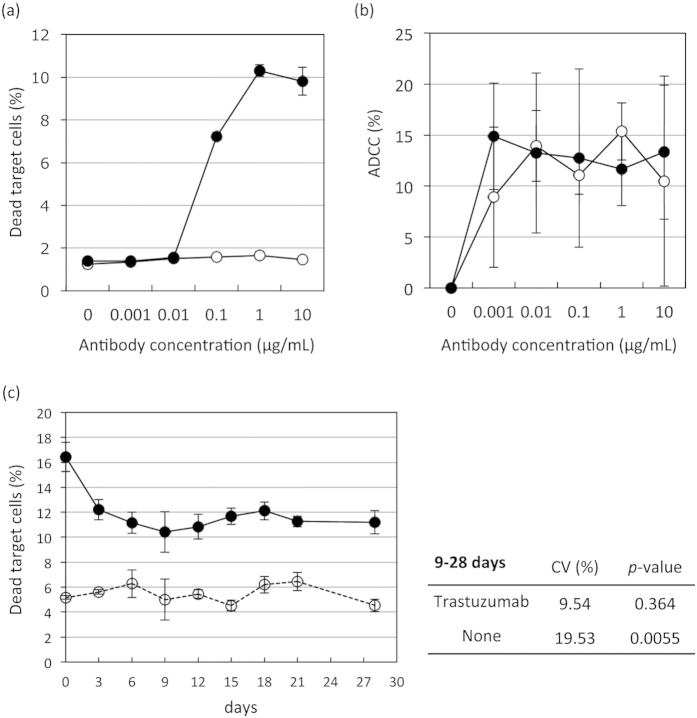
Assessment of the ADCC bioassay using frozen NK cell lines and human PBMCs. (**a,b**) BT-474 serving as target cells were incubated with frozen NK92MI + CD16a serving as effector cells at an E:T ratio of 4:1 without (white circles) or with Trastuzumab (black circles). ADCC activity was then detected using the flowcytometric (**a**) or the calcein-release (**b**) assay. (**c**) The stability of frozen human PBMCs was assessed using the flowcytometric assay. PBMCs were isolated and cryopreserved at –80˚C using CellBanker I. Portions of the PBMCs were then thawed every 3 days after initially being frozen. ADCC activity was detected, using BT-474 as target cells at an E:T ratio of 4:1, without (white circles) or with 10 μg/mL of Trastuzumab (black circles). The results of the analyses of dead target cells (%) are presented as mean values ± SD. CV values were calculated using the following formula: SD/mean×100 (%).

With the aim of expanding the versatility of this novel flowcytometric assay, we assessed the ADCC bioassay using frozen PBMCs. First, we tested frozen NK92MI + CD16a, stored in CellBanker I at −80˚C, co-cultured with BT-474 as the target tumor cells at an E:T ratio of 4:1. Our new flowcytometric assay detected ADCC activity even when frozen NK92MI + CD16a cells were used (Fig. 6a), while the calcein-release assay detected no activity (Fig. 6b). Moreover, we tested the effect of cryopreservation of freshly isolated human PBMCs, stored in CellBanker I at −80 °C. Portions of the PBMCs were thawed every 3 ~ 4 days after being frozen, and the ADCC bioassays were performed at an E:T ratio of 4:1 for 24h, followed by flowcytometric analysis. As shown in Fig. 6c, ADCC activity in Trastuzumab-treated cells gradually decreased over the course of approximately one week (Fig. 6c and Table S5, Figure S4: the data for PBMCs isolated from other donors). The differences in dead target cells (%) between Trastuzumab-treated and non-treated cell populations were also decreased, but these activities stabilized thereafter and remained essentially unchanged for periods of 12–28 days (*P* < 0.0001, Table S5). Moreover, the means of the dead target cells (%) in the Trastuzumab-treated cells did not vary significantly among time periods ranging from 9 to 28 days after being cryopreserved (*P* = 0.364, Fig. 6c). On the other hand, the means of the dead target cells (%) in the non-treated cells differed significantly among time periods (in days) (*P* = 0.0055, Fig. 6c), reflecting the tendency for ADCC activities to vary over time. There was no linear relationship between number of days and the means of the dead target cells (%) (*P* = 0.9537 in the Trastuzumab-treated and *P* = 0.8987 in the non-treated cells). Moreover, the values of the coefficient of variation (CV), defined as the ratio of the standard deviation (SD) to the mean, were 9.54 and 19.53, respectively, for Trastuzumab-treated and untreated cells. In general, CV under 20% was used as a quality check and was thus included in each analysis for acceptance.

In summary, PBMCs can be stored at −80 °C for approximately one month, allowing for sample transfer from multicenter clinical study sites to a central laboratory for analysis of ADCC using the novel bioassay described herein.

## Discussion

Herein, we established a novel non-radioactive ADCC assay allowing analysis of human PBMCs, for the purpose of immune monitoring of ongoing clinical trials. This new method was developed, employing pre-culture immunostaining and multi-parameter flowcytometry, in order to study ADCC. Our approach has several important advantages over older methods. It provides visualization of both dead and live populations of target and effector cells, and immunophenotyping at the single-cell level is also possible with simultaneous staining. The major advantages of this assay are that it can easily generate stable, reproducible and reliable data, it is operator independent, and employs the same cell number scale as the conventional release assay (Table 1). Moreover, with this assay, since CFSE-labeled target cells are cultured under adherent conditions, a low background level and long-term co-culture can be maintained, allowing evaluation of not only short-term but also long-term effects on cytotoxicity.

The challenge of performing multi-center trials, requiring sample transfer to a central laboratory for analysis, has yet to be overcome. However, freshly isolated PBMCs are widely used as effector cells in functional assays designed to evaluate ADCC. Previous studies with brief or no incubation after thawing of frozen PBMCs show decreased cytotoxic function against NK cells^16^,^17^,^18^. Recently, several studies have shown the cytotoxicity of cryopreserved PBMCs to be detectable, although the cytotoxic function of NK cells was reportedly decreased^19^,^20^. However, our new method was demonstrated to detect ADCC activity more sensitively than the Cr^51^-assay, with individual ADCC activities being measureable even when frozen PBMCs were used. On the other hand, the phenotype of frozen PBMCs was reportedly changed^21^,^22^, or minimally altered within 2 months after being frozen^23^,^24^. Indeed, Mata *et al.* suggested that PBMCs thawed and left overnight had high ADCC activity, though a change in the NK phenotype was observed after overnight incubation. Our goal is to monitor the original individual phenotype of each patient, by using frozen PBMCs immediately after thawing to perform the ADCC assay. The results described herein indicate that these novel flowcytometric methods allow very stable detection of ADCC activity, even in cells frozen for 1–4 weeks. Furthermore, the CV value was under 20% indicating reproducibility over time. Increasing the E:T ratio to approximately 10 is anticipated to enhance the stability of the data.

At present, a standardized ADCC assay for clinically monitoring ADCC activity is lacking. To date, the use of ADCC activity as a surrogate endpoint in clinical trials has been limited^2^. The stability of our new assay may enable clinical researchers to relate clinical responses to the immune responses of patients.

Recent lines of evidence suggest immune suppressor cells, such as regulatory T cells and myeloid-derived suppressor cells, to inhibit both NK cell function and ADCC activity^25^,^26^,^27^. It would thus be worthwhile to attempt immune monitoring employing multicolor flowcytometry (e.g. examining the numbers of NK cells, monocytes, and granulocytes, the expressions of activation markers, numbers of immune suppressor cells, regulatory T cells, myeloid derived suppressor cells, and so on) and relating the data obtained to ADCC activity in individual patients with advanced malignancies. Furthermore, by using our ADCC assay, in combination with pharmacokinetic and genetic analyses, we will endeavor to monitor patients currently treated in clinical trials with antibody-based drugs in order to work towards the establishment of a set of biomarkers potentially predicting clinical responses.

## Methods

### Cells

Human breast cancer cell lines, BT-474, MCF7, and Hs578T, were obtained from ATCC (Manassas, VA). These cell lines were cultured in RPMI-1640 supplemented with 10% heat-inactivated fetal bovine serum (FBS), 100 U/ml penicillin and 0.1 mg/ml streptomycin (Invitrogen Ltd, Paisley, UK). The human NK-92MI cell line was purchased from ATCC and was maintained in MyeloCult H5100 medium (STEMCELL, Vancouver, Canada). The NK-92MI cell line exogenously expressing FcγRIIIa (CD16a) was a gift from Dr. Fumiaki Koizumi (Komagome Hospital, Tokyo, Japan)^15^. All cell lines were maintained at 37 °C in a humidified atmosphere containing 5% CO_2_.

### Analysis of surface markers on breast cancer cell lines by flowcytometry

Cell surface antigen expressions were analyzed using flowcytometry. The cells were incubated with anti-HER2-PE (clone; #191924, isotype; mouse IgG2b, R&D Systems, MN), anti-NKG2D-APC (clone; 1D11, isotype; mouse IgG1κ, BD Biosciences, Heidelberg, Germany), anti-Tim3-APC (clone; #344823, isotype; rat IgG2a, R&D systems) for 30 min at 4 °C. The cells were then washed and analyzed using an EC800 flowcytometer (SONY, Tokyo, Japan).

### Isolation of PBMCs

Peripheral blood samples were obtained from 5 healthy donors (1 female and 4 males). Informed consent was obtained from all donors before the analysis. This study was approved by the institutional review board and independent ethics committee of our institute (National Cancer Center Institutional Review Board). Written informed consent was obtained from all of the healthy donors. PBMCs were separated by density-gradient centrifugation using a lymphocyte separation medium (LSM; MP Biomedicals, LLC, Solon, OH). In brief, 20 mL of two-fold diluted peripheral blood from healthy donors were layered on 15 mL of LSM and centrifuged at 400 x g for 30 min at room temperature. PBMCs were collected, and then washed 2 times with RPMI-1640 medium. After washing with the culture medium, the cells were immediately subjected to the ADCC assay and/or cryopreserved in CellBanker I at −80 °C until use.

Before use, a portion of the frozen PBMCs was thawed quickly (37 °C), washed once in 10 mL of complete culture RPMI-1640 medium, and immediately subjected to the ADCC assay.

All of the experiments were carried out in accordance with the Ethical Guidelines for Medical and Health Research Involving Human Subjects by the Ministry of Health, Labor and Welfare (MHLW) and the Ministry of Education, Culture, Sports, Science and Technology (MEXT) of Japan.

### Flowcytometric ADCC analysis

In order to evaluate patient-specific Trastuzumab-mediated ADCC, we employed our novel assay method using flowcytometry. We used BT-474 human breast cancer cell lines as the target cells and PBMCs or NK92MI + CD16a as the effector cells. BT-474 was labeled with carboxyfluorescein succinimidyl ester (CFSE) dye (Dojindo, Japan) for 5 minutes, followed by incubation overnight in a 96-well culture plate at 37 °C. ADCC was initiated by the addition of NK92MI + CD16a or human PBMCs as effector cells (1–8:1 as the effector: target (E:T) ratio) and Trastuzumab (0.001–10 μg/mL). The plate was further incubated overnight at 37˚C (5% CO_2_, humidified atmosphere). The cells were harvested, and stained with Fixable Viability Dye (FVD, eBioscience, CA) which stains dead cells. After 20 minutes of incubation, the cells were washed with 1 X phosphate buffered saline containing 2% FBS, and then subjected to FACS analysis using an EC800 (SONY, Japan).

The mean percentage ± standard deviation (SD) of each condition was calculated from 3 ~ 6 replicate wells.

### ADCC analysis using Calcein-release assay

ADCC was also examined using a calcein-acetyoxymethyl (Calcein-AM; Dojindo) release assay. The target cells were labeled with Calcein-AM for 30 min, then washed and plated onto 96-well plates at a density of 1 × 10^4 ^cells/well. Trastuzumab or Bevacizumab was added at various concentrations from 0.001 to 10 μg/mL, and fresh or frozen NK92MI + CD16a were added as effector cells at an E:T ratio of 1:1 or 4:1, respectively. The plates were then incubated for 4h at 37 °C, and the supernatants were analyzed using fluorometry to measure calcein release (cell death). For maximal release, the cells were lysed with 0.1% Triton X-100. The fluorescence value of the culture medium background was subtracted from that of the experimental release (A), the target cell spontaneous release (B), and the target cell maximal release (C). The cytotoxicity and ADCC percentages for each plate (in triplicate) were calculated using the following formulas:

Cytotoxicity (%) = (A − B)/(C − B)×100

ADCC (%) = Cytotoxicity (%, with antibody) – Cytotoxicity (%, without antibody)

For each experiment, measurements were conducted in triplicate using three replicate wells. Each experiment was repeated at least 3 times.

### Statistical analysis

Experimental data are presented as means plus/minus SD. The *t*-test was used to compare the means of the dead target cells (%) in the flowcytometric assay at each of the five antibody concentrations tested with that in the non-treated condition. In the calcein-release assay, ADCC (%) was measured as the difference between the paired values of cytotoxicity (%) with and without antibodies. The paired *t*-test was used for comparisons of the means of ADCC (%) between each antibody concentration and the non-treated cells. Analysis of variance was performed to compare the means of the dead target cells (%) in the flowcytometric assay among the time periods, in days. Regression analysis was used to assess the linear relationship between the number of days and the dead target cells (%). All *P*-value were two-sided and values less than 0.01 were considered to be statistically significant.

## Additional Information

**How to cite this article**: Yamashita, M. *et al.* A novel method for evaluating antibody-dependent cell-mediated cytotoxicity by flowcytometry using cryopreserved human peripheral blood mononuclear cells. *Sci. Rep.* **6**, 19772; doi: 10.1038/srep19772 (2016).

## Supplementary Material

Supplementary Information

## Figures and Tables

**Table 1:** Comparison of calcein-release assay and new flowcytometric assay.

	Calcein-release assay	Flowcytometric assay
Reproducibility	low (SD=4.78~17.34)	high (SD = 0.60 ~ 4.47)
Sensitivity	>1 μg/ml	>0.01 μg/ml
Frozen sample	Not detected	>0.1 μg/ml
