# Supplementary material for: A novel method for evaluating antibody-dependent cell-mediated cytotoxicity by flowcytometry using cryopreserved human peripheral blood mononuclear cells
Source: Sci Rep. 2016 Jan 27;6:19772. doi: 10.1038/srep19772 (PMC4728441; doi:10.1038/srep19772)
Supplement: Supplementary Information [file srep19772-s1.pdf]

Suppprementary material

A novel method for evaluating antibody-dependent cell-mediated cytotoxicity by flowcytometry using cryopreserved human peripheral blood mononuclear cells

Makiko Yamashita[1], Shigehisa Kitano[2,3], Aya Kuchiba[4], Hiroaki Aikawa[1], Noboru Yamamoto[2,3], Kenji Tamura[3,5], Akinobu Hamada[1,3]

1) Department of Clinical Pharmacology, National Cancer Center Research Institute, 5-1-1 Tsukiji, Chuo-ku, Tokyo 104-0045, Japan
2) Department of Experimental Therapeutics, National Cancer Center Hospital, 5-1-1 Tsukiji, Chuo-ku, Tokyo 104-0045, Japan
3) Exploratory Oncology Research and Clinical Trial Center, National Cancer Center, 5-1-1 Tsukiji, Chuo-ku, Tokyo 104-0045, Japan
4) Division of Biostatistics, Center for Research Administration and Support, National Cancer Center, 5-1-1 Tsukiji, Chuo-ku, Tokyo 104-0045, Japan
5) Department of Breast and Medical Oncology, National Cancer Center, 5-1-1 Tsukiji, Chuo-ku, Tokyo 104-0045, Japan

Supplementary Table 1. Summary statistics of Figure 4a and 4b

The means and s.d. of the dead target cells (%) in the flowcytometric assay (: Figure 4a)

		Antibody concentration (μg/ml)					
		0	0.001	0.01	0.1	1	10
Trastuzumab	Mean<br>(s.d.)	3.55<br>(1.05)	5.03<br>(1.72)	18.87<br>(4.47)	25.73<br>(3.21)	25.38<br>(3.08)	25.79<br>(4.35)
Bevacizumab	Mean<br>(s.d.)	3.46<br>(1.31)	3.13<br>(0.74)	2.87<br>(0.60)	3.33<br>(1.31)	3.29<br>(1.42)	3.43<br>(1.37)

The means and s.d. of the ADCC (%) in the calcein-release assay (: Figure 4b)

		Antibody concentration (μg/ml)					
		0	0.001	0.01	0.1	1	10
Trastuzumab	Mean<br>(s.d.)	0	6.20<br>(5.33)	16.10<br>(11.35)	31.68<br>(4.78)	51.44<br>(10.48)	57.62<br>(17.34)
Bevacizumab	Mean<br>(s.d.)	0	6.50<br>(7.60)	9.29<br>(11.08)	10.58<br>(8.63)	12.02<br>(7.97)	14.69<br>(12.50)

Supplementary Table 2. Comparison of non-treated and five antibody concentrations using NK92MI+CD16a as effector cells (Figure 4a and 4b)

P-values by *t*-test: the dead target cells (%) in the flowcytometric assay (: Figure 4a)

	Antibody concentration (μg/ml)					
	0	0.001	0.01	0.1	1	10
Trastuzumab	–	0.0141	**<.0001**	**<.0001**	**<.0001**	**<.0001**
Bevacizumab	–	0.4362	0.1509	0.7919	0.7443	0.9479

P-values by paired *t*-test: the ADCC (%) in the calcein-release assay (: Figure 4b)

	Antibody concentration (μg/ml)					
	0	0.001	0.01	0.1	1	10
Trastuzumab	–	0.0082	**0.0028**	**<.0001**	**<.0001**	**<.0001**
Bevacizumab	–	0.0333	0.0362	**0.0062**	**0.0019**	**0.0078**

Supplementary Table 3. Summary statistics of Figure 4c and 4d

The means and s.d. of the dead target cells (%) in the flowcytometric assay (: Figure 4c)

		Antibody concentration (µg/ml)					
		0	0.001	0.01	0.1	1	10
Trastuzumab	Mean<br>(s.d.)	3.43<br>(0.28)	3.67<br>(0.49)	3.46<br>(0.45)	3.92<br>(0.17)	7.12<br>(0.42)	7.22<br>(0.67)
Bevacizumab	Mean<br>(s.d.)	3.32<br>(0.32)	3.34<br>(0.47)	3.68<br>(0.36)	3.55<br>(0.55)	3.64<br>(0.35)	3.48<br>(0.45)

The means and s.d. of the ADCC (%) in the calcein-release assay (: Figure 4d)

		Antibody concentration (µg/ml)					
		0	0.001	0.01	0.1	1	10
Trastuzumab	Mean<br>(s.d.)	0	7.83<br>(12.32)	10.78<br>(7.93)	18.56<br>(12.14)	34.55<br>(16.51)	38.88<br>(10.51)
Bevacizumab	Mean<br>(s.d.)	0	7.61<br>(9.64)	8.82<br>(10.68)	6.80<br>(12.67)	5.76<br>(7.93)	11.81<br>(13.74)

Supplementary Table 4. Comparison of non-treated and five antibody concentrations using fresh PBMC as effector cells (Figure 4c and 4d)

P-values by *t*-test: the dead target cells (%) in the flowcytometric assay (: Figure 4c)

	Antibody concentration (μg/ml)					
	0	0.001	0.01	0.1	1	10
Trastuzumab	–	0.3672	0.9241	**0.0082**	**<.0001**	**<.0001**
Bevacizumab	–	0.9386	0.1309	0.4436	0.1634	0.5377

P-values by paired *t*-test: the ADCC (%) in the calcein-release assay (: Figure 4d)

	Antibody concentration (μg/ml)					
	0	0.001	0.01	0.1	1	10
Trastuzumab	–	0.2146	0.0287	0.0189	**0.0054**	**0.0004**
Bevacizumab	–	0.1379	0.1242	0.2835	0.1656	0.1128

Supplementary Table 5 Summary statistics of Figure 6c

The means, s.d. and differences of the Figure 6c

	days	0	3	6	9	12	15	18	21	28
none	Mean (s.d.)	5.17 (0.13)	5.62 (0.19)	6.29 (1.11)	5.00 (1.64)	5.26 (0.32)	4.52 (0.36)	6.19 (0.65)	6.46 (0.74)	4.54 (0.51)
	differences	—	-0.45	-1.12	0.17	-0.08	0.65	-1.02	-1.29	0.63
Trastuzumab	Mean (s.d.)	16.43 (1.16)	12.20 (0.82)	11.16 (0.85)	10.42 (1.63)	11.79 (1.18)	11.41 (0.75)	12.1 (0.72)	11.26 (0.42)	11.21 (0.93)
	differences	—	4.22	5.26	6.01	4.64	5.02	4.31	5.16	5.22
differences (Trastuzumab to none)		11.26	6.59	4.88	5.41	6.54	6.89	5.93	4.81	6.67

P-values by Student's *t*-test

days	0	3	6	9	12	15	18	21	28
none	—	0.0518	0.2318	0.8927	0.7070	0.0542	0.0506	0.0528	0.1147
Trastuzumab	—	0.0137	**0.0067**	0.0131	**0.0017**	**0.0021**	**0.0005**	**0.0010**	**0.0009**
Trastuzumab vs none	**0.0002**	**0.0004**	**0.0079**	0.0294	**< .0001**	**< .0001**	**< .0001**	**< .0001**	**< .0001**

Supplementary Figure 1

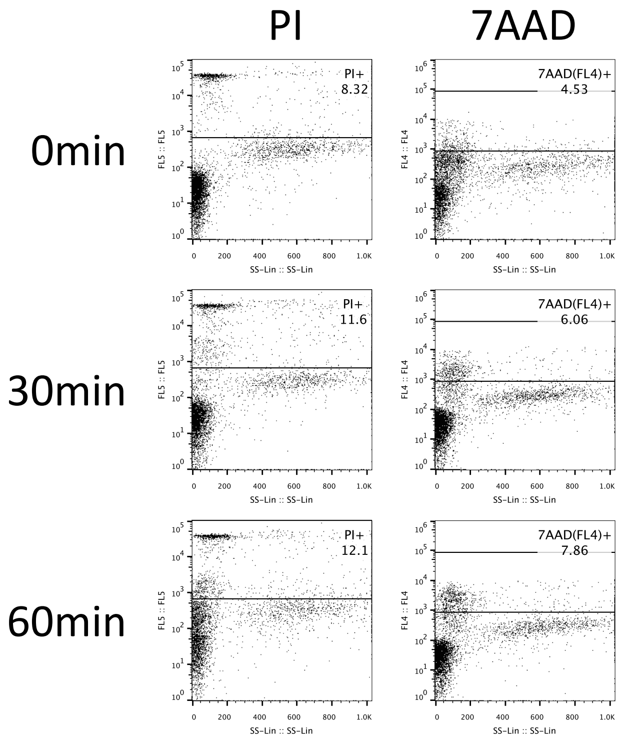

	PI	7AAD
5min	8.32	4.53
30min	11.6	6.06
60min	12.1	7.86

Figure S1. The stability of dead cell staining
Freshly isolated PBMC from healthy donors were incubated for 5 days, stained with FVD (x µg/mL, reported in Material and Method), PI (2 µg/mL, at RT for 10 min.) and 7-AAD (0.25 µg/mL, at RT for 10 min.), and then mesured by flowcytometer (EC800, SONY). The percentages of FVD+ cells were almost no changed, although PI+ cells and 7AAD+ cells were increased after staining.

Supplementary Figure 2

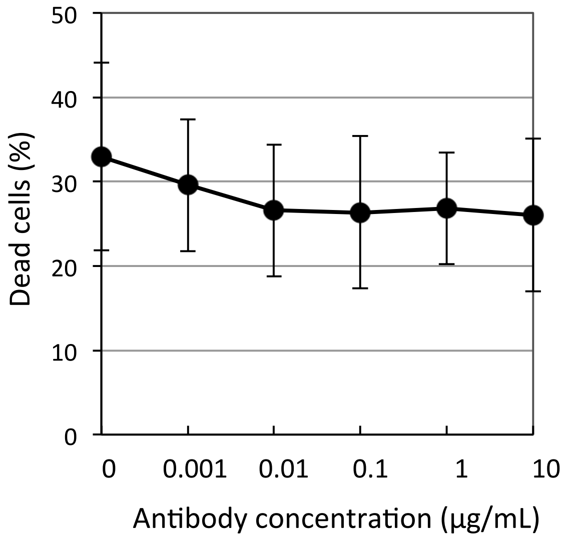

Figure S2. The effect of Trastuzumab on BT-474 cells.
The BT-474 were incubated in the presence of 0 to 10 µg/ml of Trastuzumab, without co-incubation of NK92MI+CD16a. After overnight incubation, cells were harvested and stained with FVD, and then measured employing our flowcytomtric assay. The frequency of dead cell are presented as mean values **+/-** SD of at least triplicates.

Supplementary Figure 3

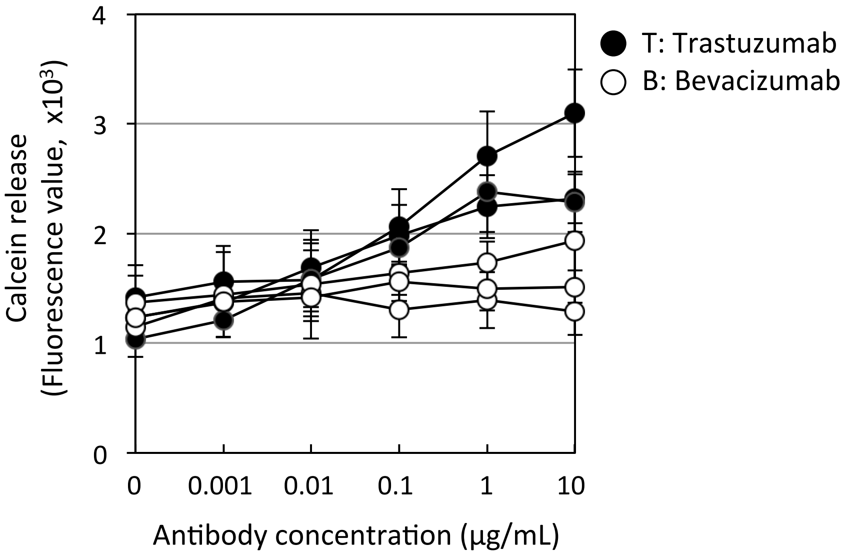

Figure S3. The Data of calcein-release analysis using fresh NK cell lines.
The BT-474 as target cells were incubated with NK92MI+CD16a in the presence of 0 to 10 µg/ml of Trastuzmab or Bevacizumab, and then measured ADCC by calcein-release assay. The experiment was repeated at least 3 times, and each experiment measured three plates in triplicate. The raw data of fluorescence value, each circle represented the mean and +/- s.d. of one experiment (9 data; three plates in triplicate in each experiment).

Supplementary Figure 4

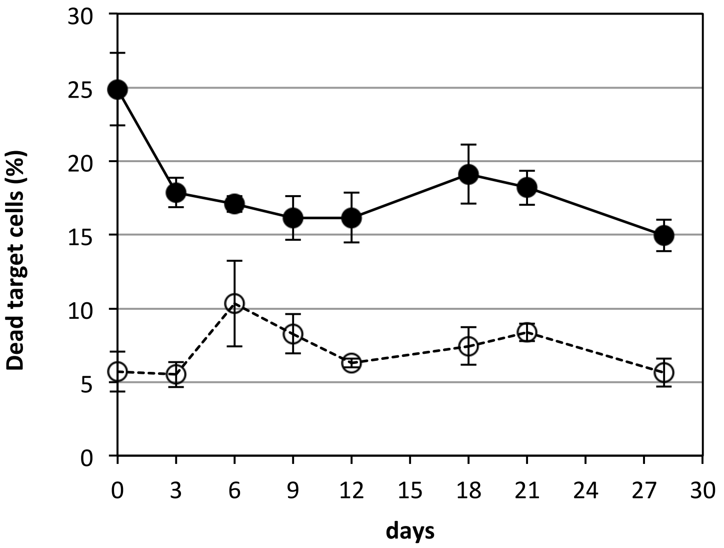

**9-28 days**	CV (%)	*p*-value
Trastuzumab	13.04	0.0315
None	20.38	0.0042

Figure S4. The Data of calcein-release analysis using fresh NK cell lines.
The stability of frozen human PBMCs was assessed using the flowcytometric assay. PBMCs from different donors of Figure 6c were isolated and cryopreserved at –80˚C using CellBanker I. Portions of the PBMCs were then thawed after initially being frozen, ADCC activity was detected, using BT-474 as target cells at an E:T ratio of 4:1, without (white circles) or with 10 µg/mL of Trastuzumab (black circles). The analyzed of dead target cells (%) are presented as mean values ± s.d.. CV value was formula: s.d. / mean x 100 (%).
